# Electrosynthetic bacterial growth under conditions simulating electric discharge in deep-sea hydrothermal fields

**DOI:** 10.1093/ismejo/wrag108

**Published:** 2026-06-23

**Authors:** Hinako Masukawa, Runa Kobayashi, Junko Watanabe, Akiko Tanizaki, Yuki Morono, Motoo Ito, Takeshi Terada, Yoshihiro Takaki, Miwako Tsuda, Yohei Matsui, Takahiro Arai, Ken Takai, Masafumi Kameya, Hiroyuki Arai, Masahiro Yamamoto

**Affiliations:** Department of Biotechnology, Graduate School of Agricultural and Life Sciences, The University of Tokyo, Tokyo 113-8657, Japan; Japan Agency for Marine-Earth Science and Technology (JAMSTEC), Institute for Extra-cutting-edge Science and Technology Avant-garde Research (X-star), Yokosuka 237-0061, Japan; Japan Agency for Marine-Earth Science and Technology (JAMSTEC), Institute for Extra-cutting-edge Science and Technology Avant-garde Research (X-star), Yokosuka 237-0061, Japan; Department of Life and Environmental System Science, Graduate School of Nanobioscience, Yokohama City University, Yokohama 236-0027, Japan; Japan Agency for Marine-Earth Science and Technology (JAMSTEC), Institute for Extra-cutting-edge Science and Technology Avant-garde Research (X-star), Yokosuka 237-0061, Japan; Japan Agency for Marine-Earth Science and Technology (JAMSTEC), Institute for Extra-cutting-edge Science and Technology Avant-garde Research (X-star), Yokosuka 237-0061, Japan; Kochi Institute for Core Sample Research, JAMSTEC, Kochi 783-8502, Japan; Kochi Institute for Core Sample Research, JAMSTEC, Kochi 783-8502, Japan; Marine Works Japan Ltd., Yokosuka 237-0063, Japan; Japan Agency for Marine-Earth Science and Technology (JAMSTEC), Institute for Extra-cutting-edge Science and Technology Avant-garde Research (X-star), Yokosuka 237-0061, Japan; Project Team for Development of New-Generation Research Protocol for Submarine Resources, JAMSTEC, Yokosuka 237-0061, Japan; Japan Agency for Marine-Earth Science and Technology (JAMSTEC), Institute for Extra-cutting-edge Science and Technology Avant-garde Research (X-star), Yokosuka 237-0061, Japan; Marine Works Japan Ltd., Yokosuka 237-0063, Japan; Japan Agency for Marine-Earth Science and Technology (JAMSTEC), Institute for Extra-cutting-edge Science and Technology Avant-garde Research (X-star), Yokosuka 237-0061, Japan; Department of Biotechnology, Graduate School of Agricultural and Life Sciences, The University of Tokyo, Tokyo 113-8657, Japan; Collaborative Research Institute for Innovative Microbiology, The University of Tokyo, Tokyo 113-8657, Japan; Department of Biotechnology, Graduate School of Agricultural and Life Sciences, The University of Tokyo, Tokyo 113-8657, Japan; Collaborative Research Institute for Innovative Microbiology, The University of Tokyo, Tokyo 113-8657, Japan; Japan Agency for Marine-Earth Science and Technology (JAMSTEC), Institute for Extra-cutting-edge Science and Technology Avant-garde Research (X-star), Yokosuka 237-0061, Japan; Department of Life and Environmental System Science, Graduate School of Nanobioscience, Yokohama City University, Yokohama 236-0027, Japan

**Keywords:** electrosynthesis, extracellular electron uptake, deep-sea hydrothermal field, carbon fixation

## Abstract

Microbial electrosynthesis is a metabolic process in which extracellular electrons are utilized as the primary energy source for carbon fixation. While microbial electrosynthesis has been proposed as a novel concept for ecological primary production, our understanding of how such microorganisms are distributed in natural environments remains limited. In this study, we constructed a laboratory-scale electrochemical cultivation system that simulates electric discharge conditions in deep-sea hydrothermal fields. Microscopic counts revealed increased cell numbers in the electrochemical culture, and 16S rRNA gene analysis revealed a significant enrichment of a novel Thiomicrorhabdus species. Quantitative PCR confirmed proliferation and enrichment of a metagenome-assembled genome (MAG), named the SREC-4. Electrochemical cultivation with ^13^C-labeled CO₂ as a substrate indicated significant ^13^C incorporation specifically in *Thiomicrorhabdus* cells including MAG SREC-4. The genome of MAG SREC-4 revealed the possession of the putative extracellular electron uptake pathway in addition to the autotrophic sulfur-oxidizing aerobic respiration pathways typically found in *Thiomicrorhabdus* members. The putative extracellular electron uptake pathway was found in a phylogenetic clade in *Thiomicrorhabdus* mainly formed by strains derived from hydrothermal fields. These results provide the direct experimental evidence from enrichment cultures derived from hydrothermal fields that an organism inhabiting deep-sea hydrothermal fields can grow electrosynthetically, and suggest that this ability is shared by other *Thiomicrorhabdus* species, specifically those found in similar environments. This finding suggests electrosynthetic growth may be widely distributed in *Thiomicrorhabdus* populations dwelling in deep-sea hydrothermal fields, the largest natural electrogenic environment on Earth.

## Graphical abstract

**Figure:**
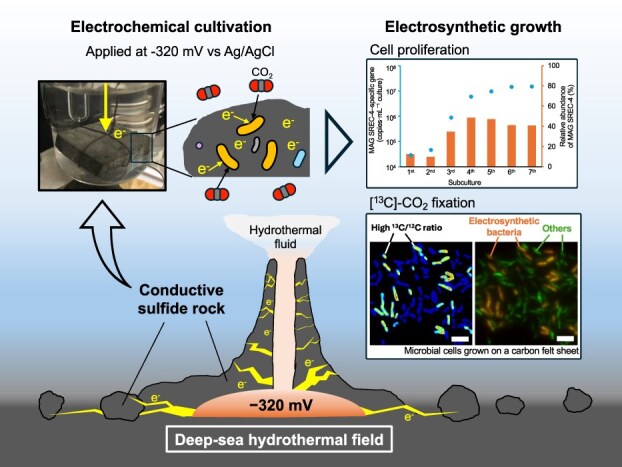

## Introduction

Electroactive microorganisms (EAMs) are the organisms capable of directly exchanging electrons with extracellular solid materials through extracellular electron transfer (EET) pathways [1]. These microorganisms have been found in various environments such as rice paddy soil, wastewater, seawater, and marine sediment, indicating their widespread presence in natural environments [2]. EAMs are also phylogenetically diverse, with representatives in all three domains of life. Perhaps include the number of phyla in each domain as well to highlight the fact that it is not just one or two organisms in each, but this ability is widespread across the tree of life [3, 4].

EAMs are typically divided into two functional groups according to the direction of electron flow: electrogens and electrotrophs. Electrogens release electrons to extracellular acceptors such as metal oxides and anodic electrodes [5]. Especially, *Shewanella oneidensis* and *Geobacter sulfurreducens* are known as the representative electrogens and the metabolic mechanisms in their EET pathways have been extensively characterized [6–8]. In contrast, electrotrophs acquire electrons from extracellular electron donors, such as reduced minerals and cathodic electrodes via the EET pathway. Their EET pathway is called the extracellular electron uptake (EEU) pathway and transport the electrons to the respiratory chain [9–11]. When energy conservation via the EEU pathway is coupled to CO_2_ fixation activity, this process is known as electrosynthesis. Electrosynthetic biomass production is attracting attention not only from an ecological perspective but also from an application standpoint due to its ability to directly utilize electrical energy [12]. Previous studies have reported methane production by methanogens (e.g. *Methanosarcina barkeri* and *Methanosarcina horonobensis*) [13] and acetate production by acetogens (e.g. *Sporomusa ovata*) [14] from cathodic biofilms. Stable electroautotrophic growth has also been demonstrated, for instance by “*Candidatus* Tenderia electrophaga” maintained on carbon felt cathodes [15]. Although other species such as *Acidithiobacillus ferrooxidans* and *Rhodopseudomonas palustris* have also been shown to grow under electrosynthetic conditions, their production rate remains quite limited [16, 17]. Many of these microbial electrochemical studies relied on cathode potentials set under artificial conditions optimized for growth. This raises a fundamental ecological question: can electrosynthetic growth occur under redox conditions that resemble those found in natural environments? To address this, it is necessary to examine environments where natural electron transfer processes are expected to occur.

In natural environments, electrotrophic or electrosynthetic microorganisms may be ubiquitous, since conductive minerals at redox boundaries have the potential to transport electrons as geochemical batteries [18]. However, the slow supply rate of reductants or oxidants limits the discharge rate, making it difficult to identify electrosynthetic microorganisms and evaluate their metabolic activity in natural communities where they compete with heterotrophic or chemosynthetic microorganisms. Among such environments, deep-sea hydrothermal systems provide a notable example. Deep-sea hydrothermal environments are known as natural electric discharge fields [19, 20]. Pyrite- and chalcopyrite-rich seafloor are conductive and form a boundary between the reductive hydrothermal fluids below the seafloor and the oxidative seawater above the seafloor. This geobattery transports electrons from the hydrothermal fluid to the seawater. The hydrothermal fluid and seawater, as reducing and oxidizing agents, respectively, are fluidly and continuously supplied, so the amount of electricity is presumed to be greater than in other electrogenic environments such as soil and sediments [19]. This discharge field is a favorable environment for electrosynthetic microorganisms that uptake electrons directly from the electrodes [21, 22]. Indeed, a novel bacterium, “*Ca*. Thiomicrorhabdus electrophagus” ISEC-1, was enriched by in-situ electrochemical cultivation using the discharge generated from a deep-sea hydrothermal vent, and genomic analyses revealed an EEU-related gene cluster homologous to that of “*Ca*. Tenderia electrophaga” [15, 23]. Members of the genus *Thiomicrorhabdus* are typically obligate autotrophs that perform sulfur oxidation coupled to aerobic respiration, and commonly observed in sulfidic habitats including hydrothermal fields, salt lakes and marine sediments [24]. The discovery of an electrosynthetic representative in this genus suggests its wider ecological role for electrosynthesis in natural hydrothermal systems.

In this study, we performed electrochemical cultivation using sulfide rock samples as electrodes under potentials simulating natural hydrothermal conditions. We examined whether microorganisms enriched under these conditions could grow electrosynthetically and investigated their enrichment dynamics and community composition. Our findings provide experimental evidence suggesting that hydrothermal discharge environments can sustain electrosynthetic growth, offering new insights into the ecological role and distribution of natural electrosynthetic microorganisms.

## Materials and methods

### Rock samples

A highly conductive sulfide rock sample (typically in the order of 10^−3^ ~ 10^0^ Ω·m) used in this work was collected from seafloor in a deep-sea hydrothermal field, the Iheya North original site of the Mid-Okinawa Trough, during a research cruise (KR18–14 leg 2, conducted by research vessel Kairei and remotely operative vehicle Kaiko Mk-IV) [25]. Small pieces of the rock sample were cut out and used as the electrode and the microbial inoculum source of electrochemical cultivation.

### Electrochemical cultivation

Composition of the electrochemical cultivation (EC) medium based on inorganic artificial seawater was: 25.0 g of NaCl, 1.0 g of (NH_4_)_2_SO_4_, 1.5 g of MgSO_4_·7H_2_O, 0.33 g of KCl, 0.3 g of CaCl_2_, 0.2 g of K_2_HPO_4_, 0.26 g of KH_2_PO_4_, 10 mg of FeSO_4_·7H_2_O, and trace element solution (Table S1) per liter of H_2_O. Electrochemical cultivation was performed in an H-shaped glass cell (VB8; EC Frontier, Japan) separated by a Nafion membrane (Nafion 117; DuPont, DE, USA) to exchange protons between the working and counter chambers. Platinum mesh and Ag/AgCl (sat. KCl) electrodes were used as the counter and reference electrodes, respectively. A piece of sulfide rock tightly tied with a titanium wire was used as the working electrode and microbial inoculum source (Fig. S1A). A carbon felt sheet tied with a titanium wire was also used as the working electrode (Fig. S1B). As a pretreatment before cultivation, the H-shaped glass cells, carbon felt sheet and titanium wires were heated in an electric furnace at 400°C for 4 hours to remove organic substances. The Nafion membranes were pretreated as previously reported [26]. All parts of the electrochemical chamber except for the working electrode were sterilized by autoclaving at 121°C for 20 min. The sulfide rock electrode was used without sterilization for the first electrochemical cultivation because it also served as the inoculum source. For the subsequent subcultures, another sulfide rock electrode was used after sterilization in an autoclave. The subculturing procedure used for electrochemical cultivation is summarized schematically (Fig. S1C). 50 ml of EC medium were poured into each glass cell, and 10% (w/v) NaHCO₃ solution was added to a final concentration of 0.1%. The medium was then bubbled with a mixed gas (N₂:O₂:CO₂ = 89:1:10), resulting in a pH of 6.2. The electrochemical cell was placed in a CO₂ incubator (WB-203 M; WakenBtech, Japan) at 15°C with 10% CO₂. A potential of −0.32 V (vs. Ag/AgCl) was applied to the working electrode, and electric currents were monitored using a potentiostat (ECstat-101 or ECstat-302, EC Frontier). The medium was replaced weekly with the same electrode. Microorganisms attached to the electrode surface served as the inoculum for subsequent cultivation. The collected spent medium was used for subsequent experiments. The overall timeline of subculture series was summarized schematically (Fig. S1D).

### Total cell counting

Microbial cells in the culture medium were fixed by 4% formaldehyde. The cell number was determined by direct cell counting using fluorescence microscopy after trapping on a membrane filter and staining with 4′,6-diamidino-2-phenylindole (DAPI) [27].

### Microbial community structure analysis based on the 16S rRNA gene

To retain electrode-attached cells as the inoculum, cells were not completely detached from the electrodes; instead, before collection of the culture medium, the electrodes were shaken in the medium to dislodge a fraction of the cells. Detached planktonic microbial cells were collected from the culture medium by filtration with a polycarbonate membrane (Isopore, 0.2 μm pore size; Merck). The extracted DNA was quantified using a Qubit dsDNA assay kit and a fluorometer (Thermo Fisher Scientific). Sequences of the 16S rRNA gene amplicons were obtained according to the previous method [23]. The resulting sequences were analyzed using the QIIME2 v2019.4.0 pipeline (Bolyen et al. 2019 Nat Biotechnol). Amplicon sequence variants (ASVs) were assigned to taxa using the SILVA 138.1 database.

### Genome reconstruction from metagenome analysis

For metagenome analysis, the DNA samples were used to construct metagenome libraries with KAPA Hyper Prep Kit Illumina System (Illumina, CA, USA) according to the manufacturer’s protocol. Paired-end sequencing was performed using the MiSeq system (Illumina, CA, USA) with a 2 × 300 bp read length. Raw reads were sequentially processed by using Trimmomatic ver. 0.39 [28] to trim the adaptor sequences and low-quality sequences and Bowtie 2 ver. 2.3.5.1 [29] to remove PhiX sequences used as internal controls. Each dataset was assembled using CLC Genomic Workbench ver. 11 (Qiagen, Germany) with the following parameter settings: 64 bp word size, 500 bp bubble size, map read back to the contigs with 0.9 length fraction, and a 0.9 similarity fraction. The genome was binned and reassembled using an R-script-based pipeline for binning and reassembly (https://github.com/MadsAlbertsen/multi-metagenome) presented in a previous report [30]. Briefly, binning was constructed based on differences in GC content, tetranucleotide frequency, and sequencing coverage across the metagenomic contigs. The contig coverage was calculated using BBMap (Bushnell B., sourceforge.net/projects/bbmap/). Bins of interest were further used as references for the mapping of the cleaned reads. The reads mapped to the reference were then assembled de novo using SPAdes ver. 3.7.1 [31]. Generated contigs were inspected and manually corrected. Completeness and contamination of the draft genome were calculated with CheckM [32] using a set of lineage-specific genes of Gammaproteobacteria. To reconstruct a phylogenomic tree, the amino acid sequences of 33 single-copy marker genes (Table S2) were extracted from the predicted proteomes of the ISEC-1 strain, nine *Thiomicrorhabdus* species, and two *Hydrogenovibrio* species. Multiple sequence alignment was performed using MAFFT v7.312 [33] and ambiguously aligned positions were removed using trimAl v1.2 [34]. After concatenating the alignment, a maximum likelihood phylogenomic tree was inferred using RAxML v8.2.9 [35] with the LG4X + G model and 100 bootstrap replications. A best-fit amino acid substitution model was chosen using AMINOSAN v1.0 [36]. A phylogenetic tree of the genus *Thiomicrorhabdus* was constructed using genes shared across all genomes registered in the Genome Taxonomy Database (GTDB), including metagenome-assembled genomes (MAGs). The tree was generated by the maximum likelihood method and visualized with the free software FigTree ver. 1.4.4 (http://tree.bio.ed.ac.uk/software/figtree/) [37]. The average nucleotide identity (ANI) and average amino acid identity (AAI) of the sequenced relatives of the genus *Thiomicrorhabdus* were estimated using OrthoANI ver. 1.8.0_121 [38] and CompareM ver. 0.1.2 (https://github.com/dparks1134/CompareM), respectively.

### Quantitative PCR

Based on the MAG SREC-4, we constructed a pair of DNA primers (Forward: 5′-CTTGTAGGGATGGAGCAGGAAA-3′, Reverse: 5′-CCACTCACCACTTACACTCCAA-3′) targeting a partial sequence of a single-copy 11-heme cytochrome *c* gene specific to the EEU pathway (Fig. S2A). This gene was selected as a quantitative PCR (qPCR) target because its unusually high heme content (11 hemes) distinguishes it structurally from other cytochrome c genes in the cluster, ensuring target specificity. For the qPCR analyses, QuantStudio 3 real-time PCR system (Applied Biosystems, USA) was used. qPCR mixtures were prepared with TB Green Premix Ex *Taq* II (Tli RNaseH Plus) and ROX Reference Dye II (Takara Bio, Japan).

### Scanning electron microscopy

The overall processing method was based on previous studies [39]. A portion of the rock electrode after electrochemical cultivation was cut out and immersed in 2.5% glutaraldehyde to fix cells. The samples were washed with EC medium and fixed at 4°C for 2 hours in artificial seawater filtered with 2% osmium tetroxide. After washing with distilled water, the samples underwent conductive staining by immersion in 0.2% aqueous tannic acid (pH 6.8) for 30 minutes, washed with distilled water, and stained with 1% aqueous osmium tetroxide for 1 hour. Finally, the samples were dehydrated in a graded ethanol series and dried using a t-butanol freeze dryer (VFD-21S; Vacuum Device, Japan). Subsequently, osmium coating was performed using an osmium plasma coater (OPC80T; Filgen, Japan). Scanning electron microscopy (SEM) observation was conducted using a field-emission scanning electron microscope Quanta 450 FEG (Thermo Fisher, USA), at an accelerating voltage of 5 kV.

### Electrochemical cultivation with ^13^C-labeled CO_2_

Electrochemical cultivation was performed in the presence or absence of ^13^C-labeled CO_2_ at a potential of −0.32 V (vs. Ag/AgCl) at 15°C for two weeks. At the time of medium replacement, NaHCO_3_ solution (^13^C:^12^C = 5:95 or 99:1) was added to the EC medium (final NaHCO_3_ conc. 0.1%) in the electrochemical chamber. A mixed gas (N_2_:O_2_:CO_2_ = 89:1:10, ^13^C:^12^C = 5:95 or 99:1) filled in a 5 L aluminum bag (CCK-5; GL Science Inc., Japan) was bubbled through the medium in the electrochemical chamber via tubing.

### EA/IRMS

After electrochemical cultivation with ^13^C-labeled CO_2_ (^13^C:^12^C = 5:95), the culture medium was filtered using a polycarbonate filter (pore size 0.2 μm, GVS, Italy) to collect the microbial cells. The filter was washed twice with fresh EC medium and vortexed with EC medium to prepare a cell suspension. The cell suspension was added onto a glass filter (GF-75, ADVANTEC) placed inside a solid silver capsule (76.9800.36, LUDI SWISS), and subsequently exposed to an HCl atmosphere at room temperature for 3 hours to remove carbonate. Excess HCl was volatilized by following incubation at 60°C. The solid silver capsule including the glass filter was loaded into a flash elemental analyzer (FLASH EA 1112 Series, Thermo Fisher) and combusted. The volatile components were analyzed using gas chromatography (GC-8A, SHIMADZU, Japan) with Isotope Ratio Mass Spectrometer (DELTA plus ADVANTAGE, Thermo Fisher) to determine the total amount of ^13^C fixed in the system.

### NanoSIMS

A 10% [^13^C]-NaHCO_3_ solution was added to the electrochemical chamber during medium exchange to a final concentration of 0.1%. The medium was bubbled with a mixed gas (N_2_:O_2_:[^13^C]-CO_2_ = 89:1:10) until the pH stabilized. To prevent the diffusion of ^13^CO_2_ gas to the outside, the electrochemical chamber was connected to a 5 L gas bag filled with the same mixed gas. Electrochemical cultivation was conducted at −0.32 V (vs. Ag/AgCl) and 15°C for two weeks. After cultivation, microbial cells attached to the working electrode were detached by vortexing in the culture medium, fixed with 4% paraformaldehyde, and collected onto an indium tin oxide (ITO)-coated membrane filter. The membrane was analyzed using a NanoSIMS 50 L (AMETEK Co. Ltd., CAMECA) at the Kochi Institute for Core Sample Research, JAMSTEC [39]. Prior to analysis, samples mounted on ITO-coated polycarbonate membranes were pre-sputtered at high beam currents to remove surface contaminants and achieve stable secondary ion signals. The secondary ions (^12^C^−, 13^C^−, 12^C^14^N^−^and ^12^C^15^N^−^) were collected simultaneously at a mass resolution of 8000, sufficient to resolve ^13^C^−^ from ^12^CH^−^ and ^12^C^15^N^−^ from ^13^C^14^N^−^. Measurements employed a 1–2 pA Cs^+^ primary beam rastered over a 25 × 25 μm area (256 × 256 pixels) with a dwell time of 5 ms per pixel. Image processing was conducted using the OpenMIMS plugin [40] within the Fiji distribution [41], following the procedures described by Morono et al. (2020) [42]. Multiple scans were aligned to correct for image drift, and accumulated ion maps were generated by summing counts from all scans to improve the signal-to-noise ratio. Ratio images (^13^C/^12^C) were then calculated from the accumulated ion maps. Regions of interest (ROIs) corresponding to individual microbial cells were defined based on elevated ^13^C/^12^C signals in the NanoSIMS images, with reference to prior fluorescence microscopy for cell localization. To minimize background interference, ROIs were tightly drawn around cell-associated signals, and surrounding background areas were used to estimate baseline levels. Isotopic ratios within each ROI were calculated after background subtraction to selectively quantify isotopic enrichment in microbial cells. The membrane with microbial cells used for NanoSIMS analysis was also applied to fluorescence *in situ* hybridization (FISH) observation. In situ DNA-hybridization chain reaction (HCR)-FISH [43] was performed using two types of DNA probes targeting all bacteria and the genus *Thiomicrorhabdus* with the green fluorescent AlexaFluor488 (Thermo Fisher Scientific) and the red fluorescent AlexaFluor555 (Thermo Fisher Scientific), respectively (Table S3). The experimental procedure followed a previously published method [43], except that the cells were stained directly on the membrane filter.

### Data availability

The 16S rRNA gene amplicon sequence data and metagenomic data are available in the DNA Data Bank of Japan (DDBJ) Sequenced Read Archive under accession numbers DRR720502 and DRR720503 - DRR720516, respectively. The genome sequences of MAG SREC-4 were deposited in DDBJ/EMBL/GenBank under the following accession numbers: BAAIHF010000001 - BAAIHF010000018. These data can be found under bioproject number PRJDB35948.

## Results

### Electrochemical microbial cultivation with sulfide rock electrode

We performed electrochemical cultivation using a piece of sulfide rock collected from a deep-sea hydrothermal field as both the working electrode and the microbial inoculum. The potential of the working electrode was poised at −0.32 V vs. Ag/AgCl (−0.12 V vs. SHE), corresponding to the potential of hydrothermal fluid previously measured in the field [19]. Electricity was provided as the sole electron source, and CO₂ as the sole carbon source. After the 12th week cultivation, community analysis of the culture medium showed enrichment of the genus *Thiomicrorhabdus*, which is known as a marine autotrophic sulfur-oxidizing bacterial taxonomic group [24] (Fig. S3). This dominance further increased by 17th week. In contrast, little enrichment of this genus was observed under open-circuit potential conditions. Metagenome-assembled genome (MAG) for the dominant strain in the electrochemical cultivation was reconstructed by the metagenomic analysis with DNA extracted from the 13th-week culture. This MAG, designated as SREC-4, contained 16S RNA genes consistent with that of the enriched strain (Table S4).

**Figure 1:**
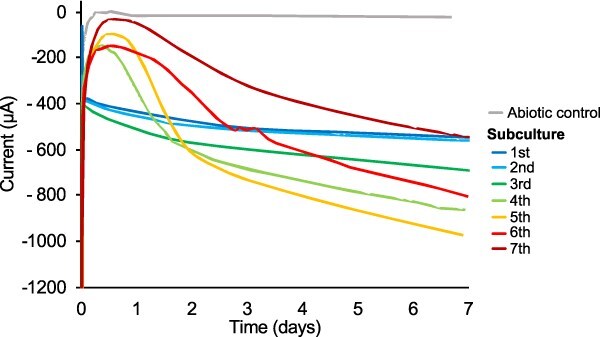
Current flow during electrochemical cultivation with sulfide rock electrode. The potential of the electrode was set at −0.32 V (vs. Ag/AgCl). Solid grey line indicates current of the first week incubation without microbial inoculation.

A subculture experiment was conducted to demonstrate the electrosynthetic growth of the dominant strain during the cultivations. The culture was transferred to a new electrochemical system, and subcultivation was repeated every 7 days and the electric currents were monitored up to the 7th week (Fig. 1). A negative current spike due to Faradaic charging of the electrode appeared in the early stages of the culture in all cases. Although the current value after the spike varied from week to week, a gradual negative current increase was observed during the 7-days culture, reaching a maximum at the 5th week. No such current was observed in the non-inoculated control, indicating that the current reflected microbial activity. Similar current profiles were reproduced in subsequent subcultures.

**Figure 2:**
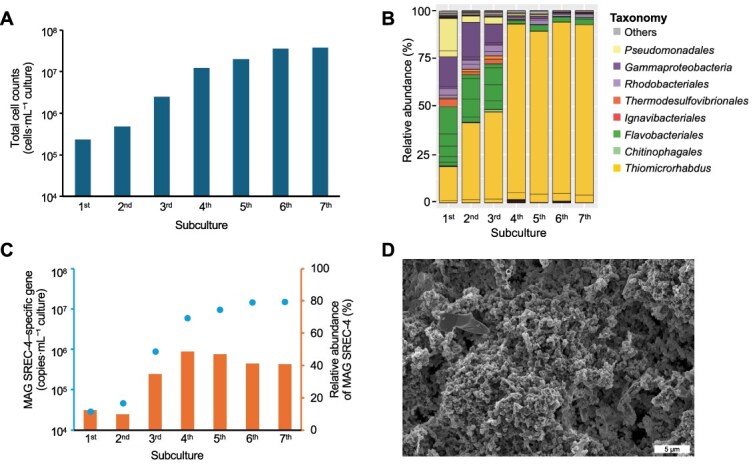
Microbial analyses of electrochemical cultivation with sulfide rock electrode; (A) total cell count during weekly electrochemical cultivation period, (B) microbial composition based on the 16S rRNA gene amplicon analysis. Solid black lines within the bars separate each ASV, (C) quantitative PCR with specific DNA primers for SREC-4. Left axis: number of copies per culture (circles), right axis: ratio of copy number to total microbial cell count (bars). (D) SEM image of the surface of the sulfide rock working electrode after cultivation.

During the seven subculture periods of the electrochemical cultivation, an increase in the total microbial cell count (biomass) in the culture medium was observed (Fig. 2A). An increase in total biomass in parallel with current value indicates that cell proliferation in this system is related to current generation. In the absence of an applied potential, the rock electrode exhibited surface corrosion in EC medium, and current could not be measured. Microbial community analysis revealed an increase in the relative abundance of the ASV corresponding to SREC-4 in the genus *Thiomicrorhabdus* as subcultivation progressed, with particularly marked dominance observed from the 4th subculture onwards (Fig. 2B). Analysis of qPCR specific to SREC-4 showed its copy number increased throughout subculturing (Fig. 2C, left axis). The relative abundance of SREC-4, as the ratio of copy number to total cell count, peaked in the 4th subculture and then stabilized at ~40% (Fig. 2C, right axis). The difference in abundance ratios between 16S rRNA and specific probes is due to differences in the number of copies (4 copies and one copy) possessed by SREC-4 (Table S4). The V4 region of the 16S rRNA gene recovered in MAG SREC-4 showed 98.9% sequence identity to the dominant *Thiomicrorhabdus* ASV over 377 bp, confirming that MAG SREC-4 represents the dominant taxon in the enrichment culture. Furthermore, SEM observation of the rock electrode surface after electrochemical cultivation revealed numerous attached cells, suggesting the establishment of an electrosynthetic community (Fig. 2D).

### Observation of carbon fixation under electrosynthetic conditions

**Figure 3:**
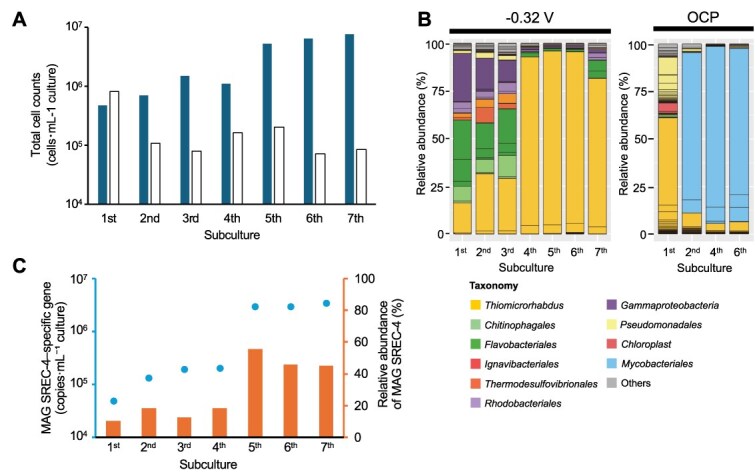
Microbial analyses of electrochemical cultivation with the carbon felt sheet working electrode; (A) total cell count during the electrochemical cultivation period, filled bars and open bars represent samples applied at −0.32 V (vs Ag/AgCl) and samples at open circuit potential, respectively. (B) Microbial composition based on the 16S rRNA gene amplicon analysis. The upper panel shows the microbial community under electrochemical cultivation at −0.32 V (vs. Ag/AgCl), and the lower panel shows the community under open circuit potential (OCP) conditions. Each ASV is separated by solid black lines within the bars, (C) quantitative PCR with specific DNA primers for SREC-4. Left axis: number of copies per culture, right axis: ratio of copy number to total microbial cell count.

Although the above experiments suggest that SREC-4 grew electrosynthetically with electricity and CO₂ as the sole electron and carbon sources, respectively, it cannot be excluded that reduced metal and sulfur ions or organic substances possibly contaminating the natural rock allowed for chemosynthetic or heterotrophic growth. We used carbon felt sheets as an alternative working electrode, which is electrochemically stable and allows the removal of organic substrates by heat treatment. Electrochemical cultivation on a carbon felt sheet electrode, performed over seven subcultures seven times showed similar results to those with the sulfide rock electrode, i.e. increase in negative current, total microbial cell count, and cell proliferation and abundance ratio of SREC-4, although the amounts of current and cell productivity were lower than those with sulfide rock electrode (Figs. 3 and S3). In addition, microbial cells were observed attaching to the surface of the carbon felt fibers after the electrochemical cultivation (Fig. S5). In the open-circuit cultivation performed for comparison, total cell numbers remained lower than those under the discharge conditions (Fig. 3A). Furthermore, the dominance of *Thiomicrorhabdus* disappeared (Fig. 3B). These results indicate that SREC-4 can grow electrosynthetically on both electrode types; sulfide rock and carbon felt sheet.

To assess the electron balance of this system, we estimated the theoretical carbon fixation supported by the measured charge (Table S5). The amount of carbon fixed during each week cultivation was estimated from the number of cells in the culture medium. The coulombic efficiency (CE) was calculated as the ratio of the charge required for carbon dioxide reduction to the current charge consumed. The maximum CEs were 0.8% and 0.9% for the rock electrode and carbon felt sheet electrode, respectively.

### Observation of carbon fixation with electrosynthetic condition

Another subculture, where SREC-4 had been enriched after the 4th week in the rock-electrode electrochemical cultivation, was cultivated for a further week under conditions containing 5% ^13^C-labeled CO₂. Following cultivation, all cells within the system were collected, and the amount of ^13^C fixation was measured using EA/IRMS. The results confirmed a higher ^13^C abundance ratio (1.62%) compared to the natural baseline (1.07%) (Table 1). Microbial composition analysis of this community based on the 16S rRNA gene showed that *Thiomicrorhabdus* ASV population accounted for 76.2% of the total bacterial 16S rRNA gene community with SREC-4 accounting for 73.8% (Fig. S6). Other bacterial ASVs were present as minor populations in the community and are primarily known to be heterotrophic; only *Thiomicrorhabdus* is capable of autotrophic growth in this community.

**Table 1:** Total carbon and ^13^C isotope ratio in the electrochemical cultivation.

Sample	Total carbon (μg)	^13^C atom%
Microbial cells with ^13^CO_2_	28.3	1.62
Microbial cells without ^13^CO_2_	16.5	1.06
Uninoculated medium with ^13^CO_2_	3.0	1.07
Uninoculated medium without ^13^CO_2_	2.3	1.07
Glass filter only	2.6	1.07

**Figure 4:**
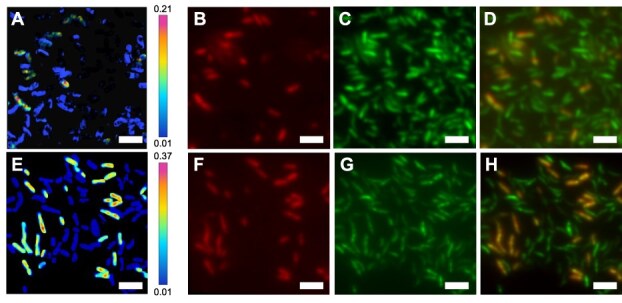
NanoSIMS and FISH images of microbial cells after electrochemical cultivation. (A, E) NanoSIMS ^13^C/^12^C ratio images. Color scale bars indicate the ^13^C/^12^C ratio. (B–D, F–H) FISH images of the same regions as in (A, E), respectively. (B, F) *Thiomicrorhabdus*-specific cells in red; (C, G) all bacteria in green; (D, H) merged images of red and green (orange cells represent *Thiomicrorhabdus* detected by both probes). (A–D) Sulfide rock electrode; (E–H) carbon felt sheet electrode. Scale bars, 5 μm. The background membrane region, which is identified by fluorescence images, is excluded from the ratio calculation and shown as black background.

We conducted electrochemical cultivation with ^13^C-labeled CO₂ again using both a sulfide rock electrode and a carbon felt sheet electrode. Subsequently, NanoSIMS analysis revealed that several microbial cells exhibited particularly high ^13^C/^12^C ratios, reaching up to 0.21 with the rock electrode (Fig. 4A) and up to 0.37 with the carbon felt sheet electrode (Fig. 4E). Notably, most of the cells with high ^13^C/^12^C ratios corresponded to *Thiomicrorhabdus* cells, as confirmed by the FISH signals (Figs. 4B–D and 4F–H).

### Characterization of MAG SREC-4

**Figure 5:**
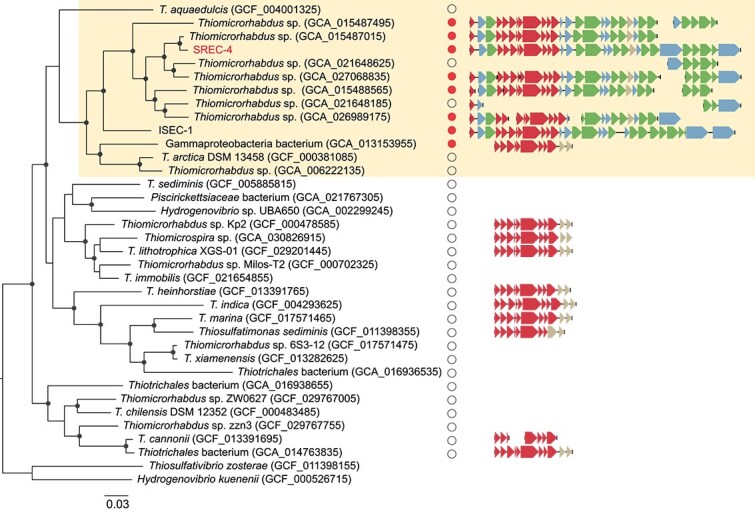
Phylogenetic genome tree of the genus *Thiomicrorhabdus* based on 33 common genes found in all *Thiomicrorhabdus* genomes registered in GTDB (Table S2). The genome tree was constructed using the genomes of 34 *Thiomicrorhabdus* species, with *Thiosulfativibrio zoserae* (GCF_011398155) and *Hydrogenovibrio kuenenii* (GCF_000526715) as outgroups. Filled and open circles indicate strains derived from deep-sea hydrothermal fields and others, respectively. Gene clusters represent the presence of homologous genes with the putative EEU pathway of strain ISEC-1. Each color represents the following: blue, multi-heme cytochrome *c* (MHC); red, cytochrome *c* maturation (Ccm) components; green, other genes; and brown, genes not found in strain ISEC-1. A boxed area highlights the clade that includes MAGs possessing the gene clusters for the putative EEU pathway.

MAG SREC-4 possessed genes for the central metabolic pathways similar to the known *Thiomicrorhabdus* species, including carbon fixation via the Calvin-Benson-Bassham (CBB) cycle, sulfur oxidation via the Sox pathway, and microaerobic respiration mediated by cbb_3_-type cytochrome c oxidase [44]. In addition, a putative EEU pathway was found, which was highly homologous to that of “Ca. Thiomicrorhabdus electrophagus” strain ISEC-1 [23], suggesting that SREC-4 possesses metabolic pathways for electrosynthesis similar to ISEC-1 (Fig. S2B). The ANI and AAI values between MAG SREC-4 and its closest relatives were all below the commonly accepted species-level thresholds (95% for both ANI and AAI). The most closely related genome to SREC-4 was a MAG (GCA_015487015), showing ANI and AAI values of 92.3% and 94.0%, respectively (Fig. S7) [45, 46]. In comparison, the strain ISEC-1, which originated from the same hydrothermal field as SREC-4 [45], showed ANI and AAI values of 78.9% and 85.5%, respectively (Fig. S7). Further analysis revealed that the gene clusters for the putative EEU pathway were also found in some other *Thiomicrorhabdus* species MAGs originated from deep-sea hydrothermal fields and were classified into a single clade in the phylogenetic tree (yellow area in Fig. 5 and Table S6). These MAGs in this clade originated from hydrothermal fields in the Pacific and Atlantic Oceans (Table S7).

## Discussion

In this work, we constructed the electrochemical cultivation system using a highly conductive sulfide rock collected from a deep-sea hydrothermal field as the working electrode, in which the redox potential of the hydrothermal fluid was poised to the electrode. This cultivation was performed under the autotrophic condition in inorganic artificial seawater with electricity as the sole electron source. A negative current consistently flowed through the working electrode (Fig. 1), reproducing the discharges observed in deep-sea hydrothermal fields. Compared to the negative control without microbial inoculation, cultivation with microbial cells generated significantly higher current, which tended to increase over weeks. This result indicated that some microorganisms on the electrode surface activated the electricity generation. Indeed, microbial mats were observed on the sulfide rock electrode surface (Fig. 2D). We observed that the number of planktonic cells in the culture increased over time (Fig. 2A). This number does not include the cells attached to the electrode surface and does not reflect the total number of cells in the entire culture system. However, the increase of the planktonic cells number (10^7^ cells/ml in 50 ml medium every week) was observed reproducibly even in the subcultures and is therefore considered to indicate the proliferation of microbial cells. Microbial community analysis showed MAG SREC-4 belonging to the genus *Thiomicrorhabdus* was enriched during the electrochemical cultivation (Fig. 2B). Furthermore, analysis of SREC-4-specific qPCR showed the proliferation of this microorganism with high dominance; up to ~50% (Fig. 2C). These results suggested substantial proliferation of SREC-4 under the electrosynthetic conditions. This system was designed to simulate the electric field conditions characteristic of deep-sea hydrothermal vent environments, therefore SREC-4 can grow electrosynthetically using electric discharge on the rock surface and may act as the primary producer in the ecosystem. In addition, enrichment and proliferation of SREC-4 were also observed using the carbon felt sheet electrode instead of the rock electrode as the working electrode (Fig. 3). Carbon felt is chemically stable and organic matter was removed from the surface by heat treatment in advance (Fig. S1B). Therefore, there was no contamination of electron sources other than electricity, more strongly supporting the electrosynthetic growth of SREC-4. It is improbable that hydrogen gas generated from the electrode is utilized as an electron source by SREC-4 in this system, given that the potential poised to the working electrode is higher than that for hydrogen generation. Furthermore, *Thiomicrorhabdus* is known to be unable to utilize hydrogen as an electron source, which is supported by the absence of oxidative hydrogenase genes in MAG SREC-4.

In this electrochemical cultivation experiment, the maximum CE was estimated at 0.9% (Table S5). Most of the remaining current electrons were likely consumed by oxygen respiration in the cultured cells. The proton motive force generated by oxygen respiration is used for ATP production via ATP synthase and NADH production via reverse electron transport (Fig. S2). A similar energy metabolic mechanism has been proposed for the autotrophic iron-oxidizing bacterium *A. ferrooxidans*, which consumed 198.1 mol of electrons from Fe^2+^ to fix 1 mol of CO₂, yielding a CE of 2.0% [47]. The lower CE observed in the electrochemical cultivation may partly reflect the consumption of organic matter by coexisting heterotrophic bacteria in the medium.

Based on the final cell yield and current consumption observed in this study, the cell production efficiency of electrosynthesis was a maximum of 1.8 × 10^6^ cells per 2 μmol electrons. This is lower than that of chemosynthesis using electron donors such as S^0^ and H_2_ (1.4 × 10^7^ cells per μmol electron donor) by *Campylobacteria* (formerly *Epsilonproteobacteria*), a dominant taxonomic group in deep-sea hydrothermal fields [48]. However, while chemosynthesis depends on the availability of specific substrates via diffusion, electrosynthesis can utilize ubiquitous redox potentials and potential gradients in the environment [6, 9], potentially functioning as an alternative even where chemical substrates are depleted or localized [49]. A 12-day *in situ* electrochemical cultivation performed near a deep-sea hydrothermal vent enriched strain ISEC-1, closely related to SREC-1, to over 10% abundance, suggesting that electrosynthesis can compete with chemosynthesis for microbial growth [23].

To investigate the CO_2_ incorporation by SREC-4, electrochemical cultivation was performed with ^13^C-labeled CO_2_ as a substrate. The total microbial cells collected from the culture showed a higher ^13^C ratio than cells cultivated without ^13^CO_2_ as well as the medium and filter controls, confirming the microbial carbon dioxide fixation (Table 1). Besides *Thiomicrorhabdus*, no other taxonomic groups known as autotrophs were detected in the microbial community (Fig. S6). Analyses of NanoSIMS and FISH showed that cells with a high ^13^C ratio were consistently *Thiomicrorhabdus* cells, confirming the clear contribution by *Thiomicrorhabdus* cells to carbon fixation with both the sulfide rock and the carbon felt sheet electrodes (Fig. 4). SREC-4 was highly predominant in this genus in the culture, meaning that SREC-4 was responsible for the carbon fixation. The results with the carbon felt sheet electrode suggest that SREC-4 utilizes electricity as the sole electron source, and the results with the rock electrode simulating natural electrical discharge strongly suggest that SREC-4 can grow electrosynthetically in deep-sea hydrothermal environments.

Our results suggest that this strain had a putative EEU pathway in addition to the characteristic features in the genus *Thiomicrorhabdus*, and was similar to the MAG of “Ca. Thiomicrorhabdus electrophagus” ISEC-1 previously enriched by *in situ* electrochemical cultivation in the same deep-sea hydrothermal field [19, 20] (Fig. S2A). According to the MAG, strain SREC-4 possesses the EET pathway and CBB cycle, suggesting that it can fix CO₂ into biomass via Rubisco (Fig. S6B). Thus, the electrosynthetic capability of strain SREC-4 is further supported by its genomic sequence information. Phylogenetic analysis of the genomes belonging to *Thiomicrorhabdus* showed that conservation of the putative EEU pathway was found within a single clade and limited to the genomes derived from deep-sea hydrothermal fields (Fig. 5, yellow area). The hydrothermal fields from which the EEU pathway-containing MAGs originate are globally widespread (Table S7). Therefore, it is plausible that an “electrosynthetic ecosystem” around hydrothermal fields may be globally distributed [5, 20, 21]. In this study, we simulated the electric discharge environment of hydrothermal fields in the laboratory and observed the enrichment and growth of *Thiomicrorhabdus* sp. by electrosynthesis. These results strongly suggest that microorganisms can utilize voltages comparable to those observed in natural environments. Furthermore, considering that weak currents are universally present in the environment, electrosynthesis may be more widely distributed than previously thought, extending beyond deep-sea hydrothermal vents. Moreover, this electrochemical cultivation system provides a new framework for handling electrosynthetic microorganisms, which have been difficult to culture, enabling systematic exploration of microbial ecosystems. While knowledge about electrosynthetic microorganisms in natural environments remains limited, this study lays the groundwork to catalyze further progress in this field.

## Supplementary Material

Supplementary_material_wrag108

## Data Availability

The DNA sequencing data generated in this study are available in the DDBJ/EMBL/GenBank databases under BioProject accession number PRJDB35948, which includes the 16S rRNA gene amplicon sequencing data, metagenomic data, and the genome sequence of MAG SREC-4. Other datasets generated and/or analysed during the current study are available from the corresponding author on reasonable request.
