# Supplementary material for: Electrosynthetic bacterial growth under conditions simulating electric discharge in deep-sea hydrothermal fields
Source: ISME J. 2026 Jun 23;20(1):wrag108. doi: 10.1093/ismejo/wrag108 (PMC13293256; doi:10.1093/ismejo/wrag108)
Supplement: Supplementary_material_wrag108 [file supplementary_material_wrag108.zip › Revised_SI_merged_wrag108.pdf]

Supplementary Materials

Electrosynthetic bacterial growth under conditions simulating electric discharge in deep-sea hydrothermal fields

Hinako Masukawa[1,2†], Runa Kobayashi[2, 3†], Junko Watanabe[2], Akiko Tanizaki[2], Yuki Morono[4], Motoo Ito[4], Takeshi Terada[5] Yoshihiro Takaki[2], Miwako Tsuda[6], Yohei Matsui[7], Takahiro Arai[5], Ken Takai[2], Masafumi Kameya[1,8], Hiroyuki Arai[1,8]*, Masahiro Yamamoto[2,3]*

Figure and Table legends

Figure S1. Electrochemical cultivation setup and experimental procedure. (A) Illustration of electrochemical cultivation system, (B) Working electrodes used in this study: sulfidic rock mineral (left) and carbon felt sheet (right), (C) Weekly medium exchange procedure, (D) Overall timeline of subculture series. Cultures were initially inoculated with rocks from a deep-sea hydrothermal vent field. Subsequent subcultures used autoclaved sulfidic rocks as working electrodes, which were replaced when precipitate accumulation impeded current flow or cell abundance decreased continuously. ^{13}C-labeling experiments for IR-MS and NanoSIMS were conducted at selected time points after sufficient growth was confirmed.

Figure S2. Putative EEU pathway gene cluster in the strain SREC-4 and the homologous gene clusters in "*Ca*. Thiomicrorhabdus electrophagus" ISEC-1 and "*Ca*. Tenderia electrophaga" (A) Each color of the gene boxes represents the following: blue, multi-heme cytochrome *c* (MHC); red, cytochrome *c* maturation (Ccm) components; green, other genes; brown, genes not found in strain ISEC-1 [1]. Numbers shown in MHC gene boxes indicate the predicted number of heme-binding sites (CXXCH motifs). The qPCR target of strain SREC-4 is indicated as black solid line. To target a 122 bp region within the coding sequence "SREC4_P0278". (B) Metabolic prediction map of strain SREC-4 based on MAG data. The structures of multiheme cytochrome c (MHC) proteins in the EET pathway were predicted by AlphaFold3. Similar to other members of the genus *Thiomicrorhabdus*, strain SREC-4 was found to possess the Sox pathway and the CBB cycle.

Figure S3. Microbial composition of the original rock–electrode batch culture based on 16S rRNA gene analysis at weeks 12th and 17th. Electrochemical cultivation was initiated using rocks collected from a deep-sea hydrothermal area as the inoculum. A potential of -0.32 V (vs. Ag/AgCl) was applied, and an open circuit

potential (OCP; no-current condition) was used as a control.

Figure S4. Current flow during electrochemical cultivation using a carbon felt sheet electrode. The abiotic control indicates that no microorganisms were inoculated.

Figure S5. Fluorescence microscope image of microbial cells attached to carbon felt fibers stained with SYBR Green. Fibers were obtained from the electrochemical cultivation system. The scale bar indicates 200 μm.

Figure S6. Microbial composition at ASV level after the electrochemical cultivation with ^{13}C-labelled CO_2 for the IR-MS analysis. Solid black lines within the bars separate each ASV.

Figure S7. Average nucleotide identity (ANI) and average amino acid identity (AAI) among genomes of strains in *Thiomicrorhabdus*. Color intensity indicates pairwise similarity, with values shown as percentages. The matrix was constructed based on the nucleotide sequences of core genes shared among *Thiomicrorhabdus* genomes registered in the Genome Taxonomy Database (GTDB).

Table S1. Trace element solution. All chemicals were of analytical grade. All chemicals were of analytical grade.

Table S2. Genes used to construct the phylogenetic tree of the genus *Thiomicrorhabdus.* These genes are shared among all members of the genus *Thiomicrorhabdus* registered in the Genome Taxonomy Database (GTDB).

Table S3. DNA probes used for FISH analysis in this study. Probe and primer sequences are shown in the 5′ to 3′ direction. Details of the FISH and PCR procedures are described in Materials and Methods.

Table S4. Information of MAG SREC-4.

Table S5. Detailed calculations for electron balance analysis.

Table S6. Information on gene clusters coding the putative EEU pathway.

Table S7. Sampling sites of MAGs containing the putative EEU gene clusters.

Reference

1. Yamamoto M, Takaki Y, Kashima H, et al. In situ electrosynthetic bacterial growth using electricity generated by a deep-sea hydrothermal vent. *ISME J* 2022;1–9. https://doi.org/10.1038/s41396-022-01316-6
2. Amann RI, Binder BJ, Olson RJ, et al. Combination of 16S rRNA-targeted oligonucleotide probes with flow cytometry for analyzing mixed microbial populations. *Appl Environ Microbiol* 1990;**56**:1919–1925. https://doi.org/10.1128/aem.56.6.1919-1925.1990
3. Yamaguchi T, Kawakami S, Hatamoto M, et al. In situ DNA-hybridization chain reaction (HCR): a facilitated in situ HCR system for the detection of environmental microorganisms. *Environ Microbiol* 2015;**17**:2532–2541. https://doi.org/10.1111/1462-2920.12745
4. Knittel K, Kuever J, Meyerdierks A, et al. *Thiomicrospira arctica* sp. nov. and *Thiomicrospira psychrophila* sp. nov., psychrophilic, obligately chemolithoautotrophic, sulfur-oxidizing bacteria isolated from marine Arctic sediments. *Int J Syst Evol Microbiol* 2005;**55**:781–786. https://doi.org/10.1099/ijs.0.63362-0
5. Simon M, Azam F. Protein content and protein synthesis rates of planktonic marine bacteria. *Mar Ecol Prog Ser* 1989. https://doi.org/10.3354/meps051201

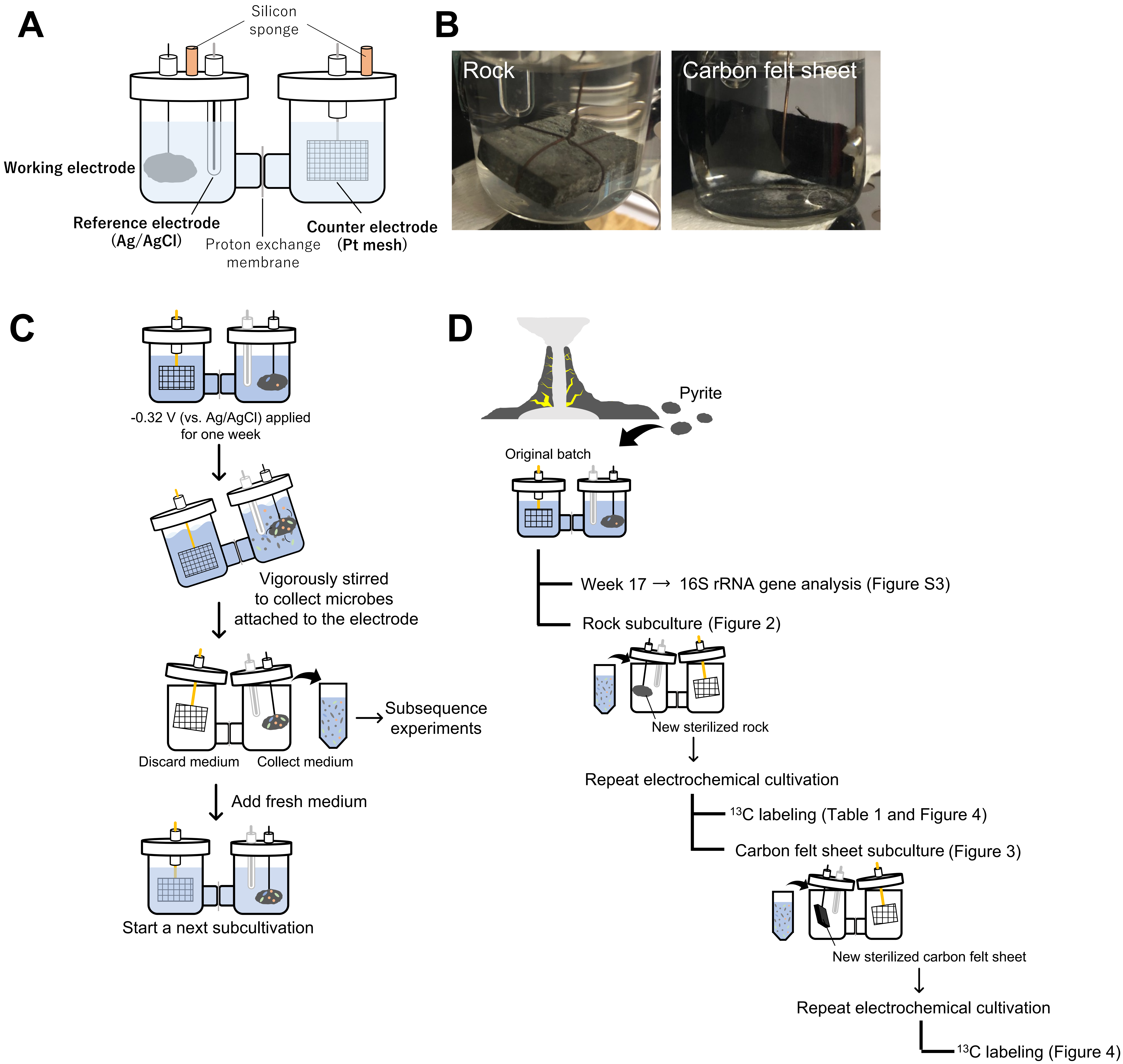
A
Silicon sponge
Working electrode
Reference electrode (Ag/AgCl)
Proton exchange membrane
Counter electrode (Pt mesh)
B
Rock
Carbon felt sheet
C
-0.32 V (vs. Ag/AgCl) applied for one week
Vigorously stirred to collect microbes attached to the electrode
Discard medium
Collect medium
Subsequence experiments
Add fresh medium
Start a next subcultivation
D
Pyrite
Original batch
Week 17 → 16S rRNA gene analysis (Figure S3)
Rock subculture (Figure 2)
New sterilized rock
Repeat electrochemical cultivation
13C labeling (Table 1 and Figure 4)
Carbon felt sheet subculture (Figure 3)
New sterilized carbon felt sheet
Repeat electrochemical cultivation
13C labeling (Figure 4)

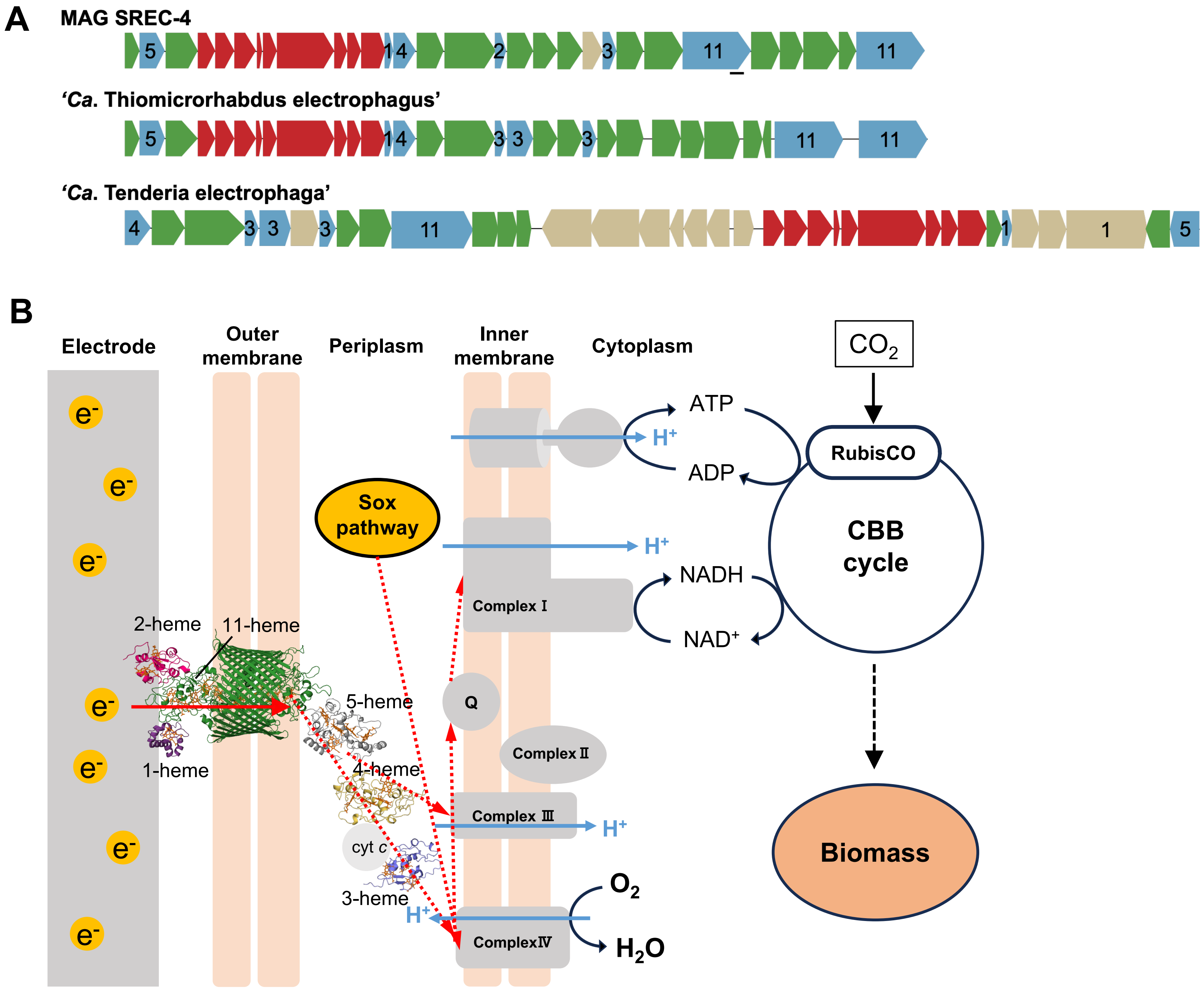
A
MAG SREC-4
'Ca. Thiomicrorhabdus electrophagus'
'Ca. Tenderia electrophaga'
B
Electrode
Outer membrane
Periplasm
Inner membrane
Cytoplasm
CO_2
RubisCO
CBB cycle
Biomass
ATP
ADP
H^+
NADH
NAD^+
Sox pathway
Complex I
Q
Complex II
Complex III
Complex IV
cyt c
2-heme
11-heme
1-heme
5-heme
4-heme
3-heme
O_2
H_2O
e^-

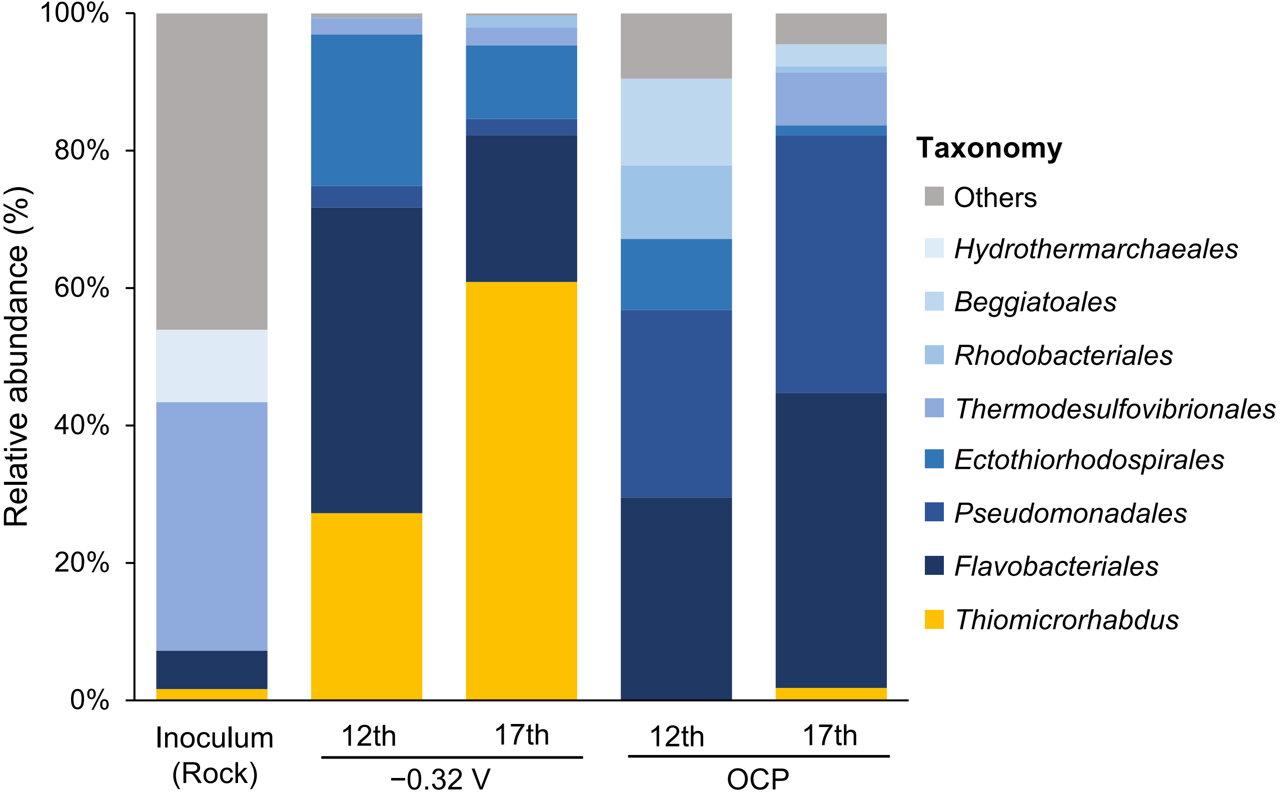

100%
80%
60%
40%
20%
0%
Relative abundance (%)
Inoculum
(Rock)
12th
17th
−0.32 V
12th
17th
OCP
Taxonomy
Others
Hydrothermarchaeales
Beggiatoales
Rhodobacteriales
Thermodesulfovibrionales
Ectothiorhodospirales
Pseudomonadales
Flavobacteriales
Thiomicrorhabdus

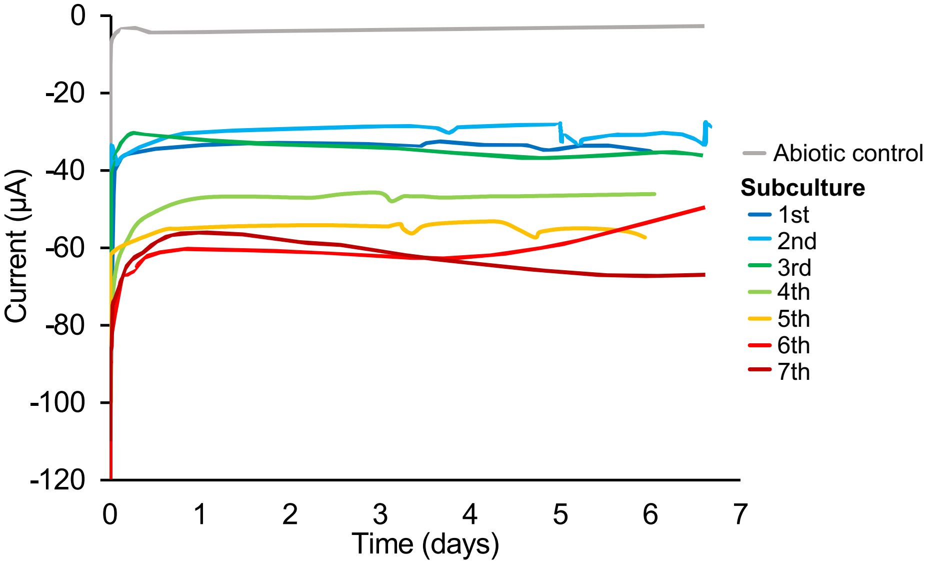
0
-20
-40
-60
-80
-100
-120
Current (μA)
0
1
2
3
4
5
6
7
Time (days)
Abiotic control
Subculture
1st
2nd
3rd
4th
5th
6th
7th

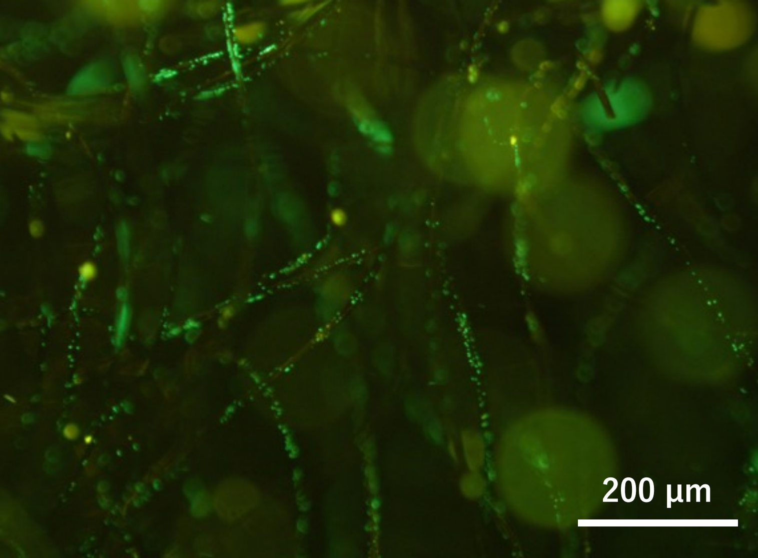
200 µm

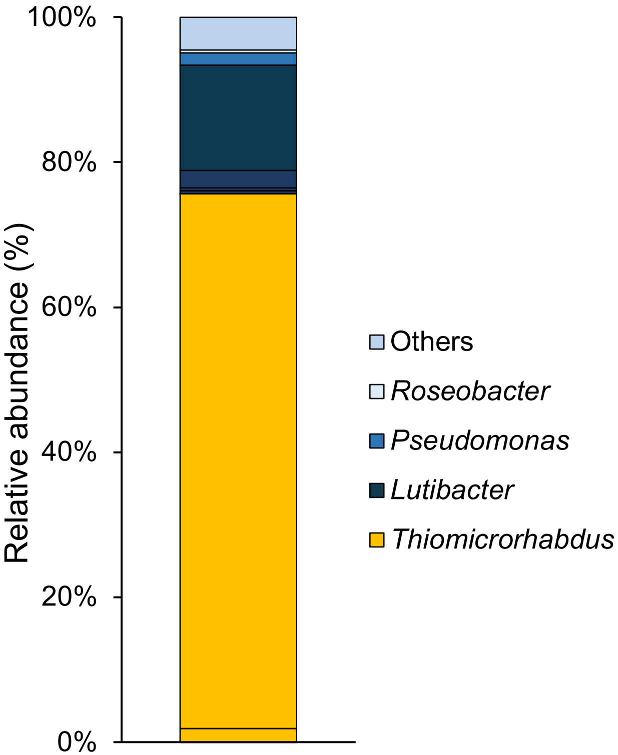
100%
80%
60%
40%
20%
0%
Relative abundance (%)
Others
Roseobacter
Pseudomonas
Lutibacter
Thiomicrorhabdus

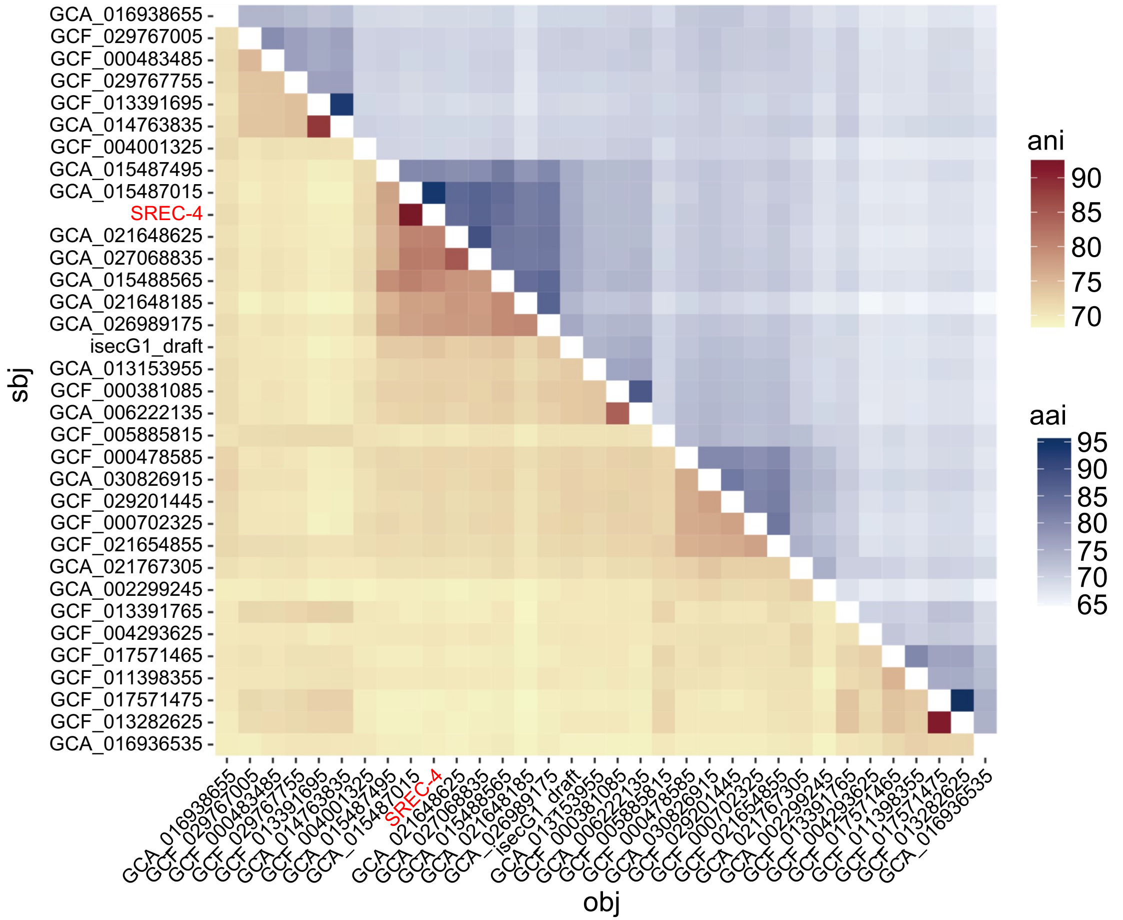
GCA_016938655
GCF_029767005
GCF_000483485
GCF_029767755
GCF_013391695
GCA_014763835
GCF_004001325
GCA_015487495
GCA_015487015
SREC-4
GCA_021648625
GCA_027068835
GCA_015488565
GCA_021648185
GCA_026989175
isecG1_draft
GCA_013153955
GCF_000381085
GCA_006222135
GCF_005885815
GCF_000478585
GCA_030826915
GCF_029201445
GCF_000702325
GCF_021654855
GCA_021767305
GCA_002299245
GCF_013391765
GCF_004293625
GCF_017571465
GCF_011398355
GCF_017571475
GCF_013282625
GCA_016936535
sbj
obj
ani
90
85
80
75
70
aai
95
90
85
80
75
70
65
