# Supplementary material for: Electrosynthetic bacterial growth under conditions simulating electric discharge in deep-sea hydrothermal fields
Source: ISME J. 2026 Jun 23;20(1):wrag108. doi: 10.1093/ismejo/wrag108 (PMC13293256; doi:10.1093/ismejo/wrag108)
Supplement: Supplementary_material_wrag108 [file supplementary_material_wrag108.zip › Table_S1_wrag108.docx]

Table S1. Trace element solution. All chemicals were of analytical grade. All chemicals were of analytical grade.

| Reagent | Added amount (g/L) |
| --- | --- |
| MnSO_4_･2H_2_O | 0.50 |
| FeSO_4_･7H_2_O | 0.10 |
| CoSO_4_･2H_2_O | 0.18 |
| CaCl_2_ | 0.10 |
| ZnSO_4_･7H_2_O | 0.18 |
| CuSO_4_･5H_2_O | 0.010 |
| KAl(SO_4_)_2_･12H_2_O | 0.020 |
| H_3_BO_3_ | 0.010 |
| Na_2_MoO_4_･2H_2_O | 0.010 |
| NiCl_2_･6H_2_O | 0.025 |
| Na_2_SeO_3_･5H_2_O | 0.0003 |
| Na_2_WO_4_･2H_2_O | 0.0004 |

Prepared with 1 L of distilled water.
