# Supplementary material for: Electrosynthetic bacterial growth under conditions simulating electric discharge in deep-sea hydrothermal fields
Source: ISME J. 2026 Jun 23;20(1):wrag108. doi: 10.1093/ismejo/wrag108 (PMC13293256; doi:10.1093/ismejo/wrag108)
Supplement: Supplementary_material_wrag108 [file supplementary_material_wrag108.zip › Table_S2_wrag108.docx]

Table S2. Genes used to construct the phylogenetic tree of the genus *Thiomicrorhabdus.* These genes are shared among all members of the genus Thiomicrorhabdus registered in the Genome Taxonomy Database (GTDB).

| Gene | Function | Average identity |
| --- | --- | --- |
| *cspA* | cold shock protein | 82 |
| *uvrA* | excinuclease ABC subunit A | 81 |
| *clpP* | ATP-dependent Clp protease, protease subunit | 90 |
| *murA* | UDP-N-acetylglucosamine 1-carboxyvinyltransferase | 81 |
| *dnaN* | DNA polymerase III subunit beta | 84 |
| *clpX* | ATP-dependent Clp protease ATP-binding subunit ClpX | 88 |
| *ABC-2.P* | ABC-2 type transport system permease protein | 82 |
| *hisD* | histidinol dehydrogenase | 88 |
| *dnaA* | chromosomal replication initiator protein | 85 |
| *infA* | translation initiation factor IF-1 | 94 |
| *rplQ* | large subunit ribosomal protein L17 | 90 |
| *trpC* | indole-3-glycerol phosphate synthase | 85 |
| *trpG* | anthranilate synthase component II | 84 |
| *gph* | phosphoglycolate phosphatase | 82 |
| *map* | methionyl aminopeptidase | 83 |
| *parC* | topoisomerase IV subunit A | 81 |
| *hpf* | ribosome hibernation promoting factor | 83 |
| *panD* | aspartate 1-decarboxylase | 82 |
| *mnmA* | tRNA-uridine 2-sulfurtransferase | 82 |
| *alaC* | alanine-synthesizing transaminase | 92 |
| *leuA* | 2-isopropylmalate synthase | 86 |
| *iscA* | iron-sulfur cluster assembly protein | 82 |
| *rlmN* | 23S rRNA (adenine2503-C2)-methyltransferase | 85 |
| *ilvB* | acetolactate synthase I/II/III large subunit | 89 |
| *leuC* | 3-isopropylmalate/(R)-2-methylmalate dehydratase large subunit | 86 |
| *fabH* | 3-oxoacyl-[acyl-carrier-protein] synthase III | 84 |
| *dksA* | RNA polymerase-binding transcription factor | 84 |
| *rplI* | large subunit ribosomal protein L9 | 85 |
| *rpsF* | small subunit ribosomal protein S6 | 85 |
| *hisG* | ATP phosphoribosyltransferase | 90 |
| *rpmF* | large subunit ribosomal protein L32 | 89 |
| *plsX* | phosphate acyltransferase | 82 |
| *fur* | Fur family transcriptional regulator, ferric uptake regulator | 87 |
