# Supplementary material for: Electrosynthetic bacterial growth under conditions simulating electric discharge in deep-sea hydrothermal fields
Source: ISME J. 2026 Jun 23;20(1):wrag108. doi: 10.1093/ismejo/wrag108 (PMC13293256; doi:10.1093/ismejo/wrag108)
Supplement: Supplementary_material_wrag108 [file supplementary_material_wrag108.zip › Table_S3_wrag108.docx]

Table S3. DNA probes used for FISH analysis in this study. Probe and primer sequences are shown in the 5′to 3′ direction. Details of the FISH and PCR procedures are described in Materials and Methods.

| Probe name | Sequence (5’–3’) | Reference |
| --- | --- | --- |
| *Initiator probe* |  |  |
| ThiomECP555 | CCAGTTATCAGTAGTCCGTCCTTCATAAAAAGGTATTAACCTTACTACCTTC | This study |
| EUB338-initiatorH | CCGAATACAAAGCATCAACGACTAGAAAAAAGCTGCCTCCCGTAGGAGT | [2] |
| *Amplifier probe* |  |  |
| H1 | TCTAGTCGTTGATGCTTTGTATTCGGCGACAGATAACCGAATACAAAGCAT | [3] |
| H2 | CCGAATACAAAGCATCAACGACTAGAGATGCTTTGTATTCGGTTATCTGTCG | [3] |
| C1 | ATGAAGGACGGACTACTGATAACTGGGACTTCCATACCAGTTATCAGTAGTC | [3] |
| C2 | CCAGTTATCAGTAGTCCGTCCTTCATGACTACTGATAACTGGTATGGAAGTC | [3] |

Underlined sequences are reverse complementary to the targeted microbial sequences (ThiomECP555: genus *Thiomicrorhabdus*, EUB338-initiatorH: all bacteria).
