# Supplementary material for: Electrosynthetic bacterial growth under conditions simulating electric discharge in deep-sea hydrothermal fields
Source: ISME J. 2026 Jun 23;20(1):wrag108. doi: 10.1093/ismejo/wrag108 (PMC13293256; doi:10.1093/ismejo/wrag108)
Supplement: Supplementary_material_wrag108 [file supplementary_material_wrag108.zip › Table_S4_wrag108.docx]

Table S4. Information of MAG SREC-4.

| Contents | MAG SREC-4 |
| --- | --- |
| Number of contig | 18 |
| Total length | 2,273,693 bp |
| N50 | 220,367 bp |
| GC content | 41.50% |
| Average coverage | 178 |
| Number of genes |  |
| CDS | 2,097 |
| Copy number of the rRNA operon | 4 |
| tRNA | 41 |
| Other RNA | 3 |
| Evaluation |  |
| Completeness | 100% |
| Contamination | 0.35 |
