# Supplementary material for: Electrosynthetic bacterial growth under conditions simulating electric discharge in deep-sea hydrothermal fields
Source: ISME J. 2026 Jun 23;20(1):wrag108. doi: 10.1093/ismejo/wrag108 (PMC13293256; doi:10.1093/ismejo/wrag108)
Supplement: Supplementary_material_wrag108 [file supplementary_material_wrag108.zip › Table_S5_wrag108.docx]

Table S5. Detailed calculations for electron balance analysis.

|  | Total cells  in 50 mL medium | Total carbon (pg) | Required charge for CO_2_ reduction (C) | Total current charge (C) | CE* (%) |
| --- | --- | --- | --- | --- | --- |
| Rock |  |  |  |  |  |
| 1st | 1.2×10^7^ | 3.22×10^5^ | 0.010 | 482.2 | 0.002 |
| 2nd | 2.4×10^7^ | 6.72×10^5^ | 0.022 | 331 | 0.01 |
| 3rd | 1.3×10^8^ | 3.50×10^6^ | 0.11 | 368.8 | 0.03 |
| 4th | 6.2×10^8^ | 1.72×10^7^ | 0.55 | 385.5 | 0.1 |
| 5th | 1.0×10^9^ | 2.80×10^7^ | 0.90 | 400.2 | 0.2 |
| 6th | 1.8×10^9^ | 4.90×10^7^ | 1.58 | 311 | 0.5 |
| 7th | 1.9×10^9^ | 5.18×10^7^ | 1.67 | 207.5 | 0.8 |
| Carbon felt |  |  |  |  |  |
| 1st | 2.4×10^7^ | 5.64×10^5^ | 0.021 | 17.9 | 0.1 |
| 2nd | 3.5×10^7^ | 8.40×10^5^ | 0.032 | 17.4 | 0.2 |
| 3rd | 7.5×10^7^ | 1.80×10^6^ | 0.068 | 19.7 | 0.3 |
| 4th | 5.5×10^7^ | 1.32×10^6^ | 0.050 | 24.9 | 0.2 |
| 5th | 2.6×10^8^ | 6.24×10^6^ | 0.23 | 28.2 | 0.8 |
| 6th | 3.2×10^8^ | 7.56×10^6^ | 0.28 | 34.4 | 0.8 |
| 7th | 3.8×10^8^ | 9.00×10^6^ | 0.34 | 35.8 | 0.9 |

*CE (Coulombic efficiency) was calculated as the ratio of the charge theoretically required for biomass carbon fixation to the total charge passed during the 7-day cultivation period. According to the stoichiometry of carbon fixation via the CBB cycle (3CO₂ + 12e^-^ + 9ATP + 6H⁺→ glyceraldehyde-3-phosphate + 9ADP + 3H₂O + 8P_i_), 4 electrons are required per carbon atom fixed.

**Cell size assumption:**

The cell was approximated as rod-shape. The radius was assumed to be *r* = 0.25 μm (diameter = 0.5 μm), and the total cell length was 1.2 μm [4]. Therefore, the cylindrical height was calculated as:

$$h = 1.2 - 2r = 1.2 - 0.5 = 0.7 \mu m$$

**Cell volume calculation:**

$$V= {\pi r}^{2}h + \frac{4}{3}{\pi r}^{3}$$

$$\boldsymbol{=}\pi\times\left( 0.25 \mu m \right)^{2}\times0.7 \mu m + \frac{4}{3}\pi\times\left( 0.25 \mu m \right)^{3}\approx0.14 {\mu m}^{3}$$

**Carbon amount per cell** [5]**:**

$${88.6\times V^{0.59}=88.6 \times0.14}^{0.59}=28 fg/cell$$

**Electrons required per cell:**

$$Moles of carbon=\frac{28\times{10}^{-15} g}{12 g \mathrm{mol}^{-1}}=2.3\times{10}^{-15} \mathrm{mol}$$

$$Electrons= 2.3 \times{10}^{-15} \mathrm{mol}\times4e^{-}/\mathrm{carbon}\times6.022\times{10}^{23} \mathrm{mol}^{-1}$$

$$=5.54\times{10}^{9} e^{-}/cell$$

**Cumulative charge calculation:**

$$Q =\int Idt$$
