# Supplementary material for: Electrosynthetic bacterial growth under conditions simulating electric discharge in deep-sea hydrothermal fields
Source: ISME J. 2026 Jun 23;20(1):wrag108. doi: 10.1093/ismejo/wrag108 (PMC13293256; doi:10.1093/ismejo/wrag108)
Supplement: Supplementary_material_wrag108 [file supplementary_material_wrag108.zip › Table_S7_wrag108.docx]

Table S7. Sampling sites of MAGs containing the putative EEU gene clusters.

| ID | Sample origin | Sampling area |
| --- | --- | --- |
| GB_GCA_015487495.1 | deep-sea hydrothermal vent | Pacific Ocean, New Zealand |
| GB_GCA_015487015.1 | deep-sea hydrothermal vent | Pacific Ocean, New Zealand |
| GB_GCA_021648625.1 | marine sediment | Off the coast, South Africa |
| GB_GCA_027068835.1 | marine hydrothermal vent | East Pacific Rise |
| GB_GCA_015488565.1 | deep-sea hydrothermal vent | Pacific Ocean, New Zealand |
| GB_GCA_021648185.1 | marine sediment | Off the coast, South Africa |
| GB_GCA_026989175.1 | deep-sea hydrothermal vent | Mid Atlantic Ridge |
| SREC-4 | deep-sea hydrothermal field | Mid Okinawa Trough, Japan |
| ISEC G1_draft | deep-sea hydrothermal field | Mid Okinawa Trough, Japan |
